# A Complex Evaluation of the In-Vivo Biocompatibility and Degradation of an Extruded ZnMgSr Absorbable Alloy Implanted into Rabbit Bones for 360 Days

**DOI:** 10.3390/ijms222413444

**Published:** 2021-12-14

**Authors:** Karel Klíma, Dan Ulmann, Martin Bartoš, Michal Španko, Jaroslava Dušková, Radka Vrbová, Jan Pinc, Jiří Kubásek, Marek Vlk, Tereza Ulmannová, René Foltán, Eitan Brizman, Milan Drahoš, Michal Beňo, Vladimír Machoň, Jaroslav Čapek

**Affiliations:** 1 Department of Stomatology, General Teaching Hospital, 1st Faculty of Medicine, Charles University, Kateřinská 32, 121 08 Prague, Czech Republic; Karel.Klima@vfn.cz (K.K.); dan.ulmann@vfn.cz (D.U.); martin.bartos@vfn.cz (M.B.); michal.spanko@vfn.cz (M.Š.); radka.vrbova@vfn.cz (R.V.); Marek.Vlk@vfn.cz (M.V.); tereza.ulmannova@vfn.cz (T.U.); rene.foltan@vfn.cz (R.F.); eitan.brizman@vfn.cz (E.B.); drahomil@gmail.com (M.D.); michal.beno@vfn.cz (M.B.); Vladimir.Machon@vfn.cz (V.M.); 2 Department of Anatomy, 1st Faculty of Medicine, Charles University, 121 08 Prague, Czech Republic; 3 Department of Pathology, 1st Faculty of Medicine, Charles University, 121 08 Prague, Czech Republic; jaroslava.duskova@lf1.cuni.cz; 4 Department of Functional Materials, FZU-The Institute of Physics of the Czech Academy of Sciences, Na Slovance 1999/2, 182 21 Prague, Czech Republic; pinc@fzu.cz; 5 Department of Metals and Corrosion Engineering, University of Chemistry and Technology, Technická 5, 166 28 Prague 6, Czech Republic; Jiri.Kubasek@vscht.cz

**Keywords:** absorbable metals, in vivo, magnesium, zinc, strontium, toxicity, biocompatibility, systemic reactions, alloy accumulation, internal organs

## Abstract

The increasing incidence of trauma in medicine brings with it new demands on the materials used for the surgical treatment of bone fractures. Titanium, its alloys, and steel are used worldwide in the treatment of skeletal injuries. These metallic materials, although inert, are often removed after the injured bone has healed. The second-stage procedure—the removal of the plates and screws—can overwhelm patients and overload healthcare systems. The development of suitable absorbable metallic materials would help us to overcome these issues. In this experimental study, we analyzed an extruded Zn-0.8Mg-0.2Sr (wt.%) alloy on a rabbit model. From this alloy we developed screws which were implanted into the rabbit tibia. After 120, 240, and 360 days, we tested the toxicity at the site of implantation and also within the vital organs: the liver, kidneys, and brain. The results were compared with a control group, implanted with a Ti-based screw and sacrificed after 360 days. The samples were analyzed using X-ray, micro-CT, and a scanning electron microscope. Chemical analysis revealed only small concentrations of zinc, strontium, and magnesium in the liver, kidneys, and brain. Histologically, the alloy was verified to possess very good biocompatibility after 360 days, without any signs of toxicity at the site of implantation. We did not observe raised levels of Sr, Zn, or Mg in any of the vital organs when compared with the Ti group at 360 days. The material was found to slowly degrade in vivo, forming solid corrosion products on its surface.

## 1. Introduction

Age-related fractures in the USA are projected to increase from 2.1 million in 2005 to over 3 million fractures in 2025 [1]. In the rest of the world, the situation will be very similar. The aging world population looks to be the most important factor causing this increase. Treatment of bone fractures in human and veterinary medicine is based on conservative (casts, splints) or surgical treatments (osteosynthesis). Surgical treatment using metallic plates and screws is the accepted form of treatment for spine, hip, knee, or facial fractures. The gold standard plate material is either medically pure titanium, titanium alloys, or stainless steel. Titanium and steel are not absorbable. To remove a non-absorbable metallic implant, the patients are forced to undergo a second surgical procedure—doubling the number of operations required. This significantly prolongs the convalescence period and also increases the costs connected with the healthcare. Absorbable, or biodegradable implants are able to degrade in the organism after fulfilling their mission, which makes the second surgical procedure unnecessary. This means that application of absorbable implants is connected not only with an increased comfort of patient life, but also with significant economical savings [2].

Biodegradable polymers, such as polyglycolic (PGAs) and polylactic acids (PLAs) and their copolymers [3] and biodegradable ceramic [4] are already used in clinical practice. Biodegradable polymers are mainly used in the fabrication of resorbable sutures or small bone implants. This is because they are highly biocompatible and degraded forming non-toxic compounds. Formation of copolymers allows to reach a wide scale of mechanical and degradation properties allowing to adjust implant characteristics to the desired values. The aforementioned polymers and their copolymers exhibit sufficient mechanical behavior for those applications, e.g., tensile strengths up to 100 MPa, Young’s moduli of several units of GPa and elongation to fracture sufficient for biomedical applications (up to 20%) [4,5,6]. Regarding orthopedic applications, biodegradable polymers are used for the fabrication of pins, anchors, screws or meniscus arrows [7]. Biodegradable ceramic are mainly used as a filler of bone defects [4,5]. There are many absorbable polymer-based implant materials and particular implants currently available in the global market (Sysorb^®^, Biosteon^®^, ProToe^®^—Endosorb^®^, Inion OTPS^TM^, LactoSorb^®^, etc. [8]); however, their application is limited to conditions with lower load forces. For load-bearing osteosynthesis applications, metallic materials possess more ideal mechanical behavioral traits (ductility, high strengths, etc.) [2]. A fully absorbable metal, therefore, could be an ideal solution for the treatment of fractures in both human and veterinary medicine, eliminating the need for a second procedure. Due to their high strength and ductility, biodegradable metallic materials seem to be ideal for the fabrication of implants for cardiovascular (stents) [9] or orthopedic [10] applications (screws, nails, splints, etc.). The design of biodegradable metallic materials is very challenging, because many aspects must be considered and some of them are practically impossible to simulate in-vitro (degradation rate and mechanism, corrosion fatigue, corrosion stress cracking, cytotoxicity, etc. [11,12,13,14,15,16,17]). Many metallic materials are even not natural to the human body and a lot of them are also toxic, the contenders must therefore be carefully chosen—based on several criteria (toxicity, corrosion, mechanical behavior, etc.), as described in our previous study [18]. Recently, three kinds of biodegradable metallic materials have been investigated. Firstly, magnesium-based materials [19,20], secondly, iron-based materials [21] and lastly, zinc-based materials [22,23]. Compared to the magnesium- and iron-based materials, the zinc-based materials show almost ideal degradation rates for implantology, especially for the fabrication of thin-wall implants, such as stents or sutures. Bowen et. al. found that thin Zn wires implanted in rat aortas degraded at a rate approaching 20 µm/year, which was recommended as the ideal one for the arterial stents [24]. Depending of the application, the biodegradable implants should fully degrade within 1–2 years, implying that small bone implants, screws and plates with a diameter or thickness of only few millimeters, have to corrode by the rate at least of several tenths a millimeter per year. The corrosion rate can be increased to more rational values by suitable alloying, particularly through the implementation of micro-galvanic cells into the material [23,25]. The corrosion process of Zn-based alloys is not connected with hydrogen evolution, which is a strong advantage compared to the Mg-based materials whose degradation can be connected with the formation of gas bubbles and pockets negatively influencing the healing process [18,26].

In our pilot in-vivo study, the ternary alloy Zn-0.8Mg-0.2Sr (wt.%) was implanted into rabbit tibia for 120 days [18]. This short-term study indicated promising behavior of the alloy for use in implantology [18]. We therefore decided to investigate the in-vivo behavior of this alloy for longer implantation periods (240 and 360 days). The in-vivo degradation, local and systemic toxicity, integration, and general biocompatibility were investigated on a rabbit model. The evolution of those characteristics with time was evaluated from the obtained results. The presented study is the next piece of a mosaic characterizing this alloy as a potential absorbable implant material [2,18,27,28]

## 2. Results

### 2.1. Results of Radiographic Examinations

**Figure 1:**
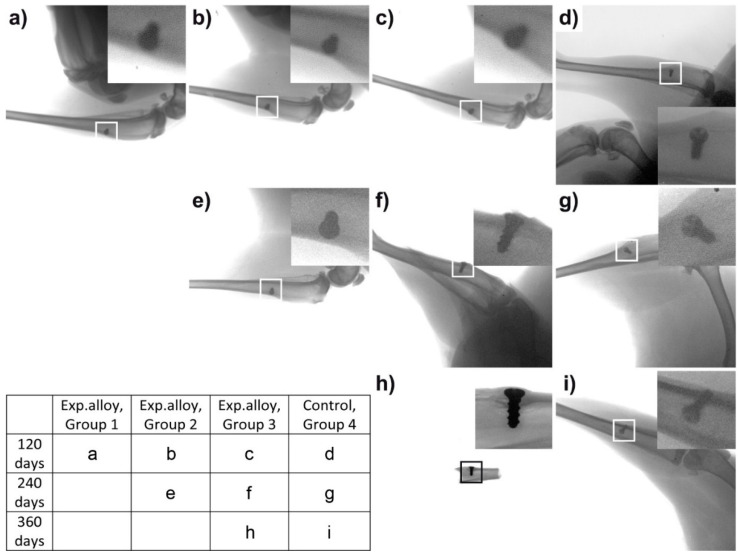
X-ray images (plain radiography) of the tibia bones of experimental rabbits. (**a**–**d**) 120 days after implantation, (**a**–**c**) experimental Zn-based screw, (**d**) control Ti-based screw; (**e**–**g**) 240 days after implantation; (**e**,**f**) experimental Zn-based screw; (**g**) control Ti-based screw; (**h**) 360 days after experimental Zn-based screw implantation; (**i**) 360 days after control Ti-based screw implantation. There were no osteolytic changes seen on the boundaries between the bone and experimental and Ti-based screws. No detectable differences between groups after 120, 240, and 360 days of implantation. No detectable changes between experimental screw groups and Ti-based screws used as a control.

The specimens were visualized utilizing a standardized 2D X-ray imaging technique in two planes 120, 240, and 360 days after implantation in all groups. The obtained pictures are shown in Figure 1. No osteolytic changes were observed, nor were there any signs of irritation to the bone adjacent to the experimental Zn-based and control Ti-based screws. We observed no differences between all groups.

### 2.2. Results of Micro-CT

**Figure 2:**
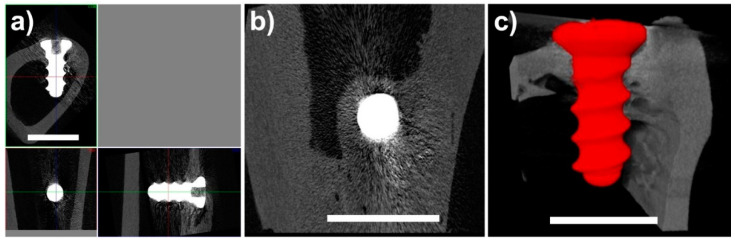
Micro-CT visualizations; (**a**) standardized cross-sectional images in 3 perpendicular planes; (**b**) implant surrounded by bone; (**c**) 3D visualization of implant 240 days after healing. Scale bar = 4 mm.

Specimens were visualized utilizing standardized 2D cross-sectional images in three perpendicular planes (Figure 2a). Implants were positioned in cortical bone and medullary cavities with the exception of two specimens that were inserted intracortically (Figure 2b) and three specimens inserted bicortically. 

**Figure 3:**
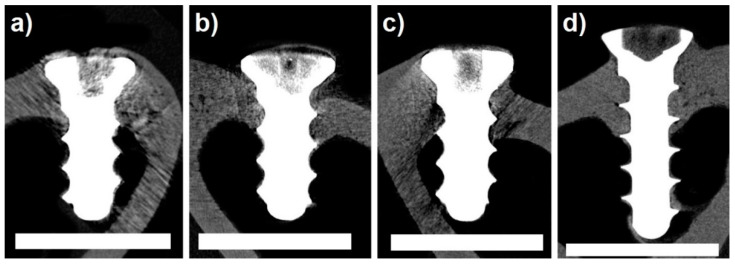
Micro-CT cross-sectional images; (**a**) tested implant, 120 days of healing; (**b**) tested implant, 240 days of healing; (**c**) tested implant, 360 days of healing; (**d**) control specimen (Ti-based screw). All specimens showed bone healing without any signs of adverse reaction. To enhance clarity, image noise was reduced except around the areas of the bone and implant. Scale bar = 4 mm.

Micro-CT image data quality enabled the quantitative evaluation of bone–implant contact (manual 2D measurement), quantitative 3D analysis of the implant structure, and qualitative evaluation of the bone surrounding the implant. However, metal-induced artefacts in the experimental groups (Groups 1–3) did not allow the quantitative 3D analysis of bone–implant contact (Figure 3). For this reason, Ti-based implants are advantageous due to their lower X-ray density.

All evaluated implants were in contact with the surrounding bone with no signs of adverse reaction (foreign body reaction or fibrointegration). The thickness of the bone surrounding the implants was higher in comparison with average cortical thickness. Even in the case of intracortical implantation (Figure 2b) when the implant diameter was higher than the cortical bone thickness (resulting in bone dehiscence after implantation), the implants were surrounded by new bone. The mean BIC values obtained through the micro-CT analyses are listed in Table 1. The differences were not significant (Mann–Whitney paired test, *p* = 0.05). Implants inserted intracotrically were excluded from this evaluation because the BIC values were influenced by implant position, bone structure (no trabecular bone in marrow cavity), and cortical bone thickness, so its importance was thus limited. This was the reason for the exclusion of two bone specimens from Group 3 from the BIC calculations (experimental alloy, 360 days). The standard deviation calculated from one specimen was zero (Table 1).

There were no signs of implant degradation over time. Only minor changes in volume and surface were found between the 120-, 240-, and 360-day healing periods (Table 2) with no statistical significance (Mann–Whitney paired test, *p* = 0.05). The expected substantial decrease in volume with an associated transient increase in surface area, as a result of material fragmentation creating new surfaces, was not observed—in contrary to our previous study [29]. It has to be mentioned, that the degradation observed by micro-CT could have been underestimated, because the metal-induced artefacts deteriorated the resolution at the implant–tissue interface. Therefore, changes in implant dimensions and the formation of corrosion products may not have been accurately detected in the region of several tens of micrometers in distance from the surface. The evaluation of artifacts around a high-density object in an X-ray is affected by a range of several pixels per micro-CT.

### 2.3. SEM–EDS of the Implant–Bone Interface

**Figure 4:**
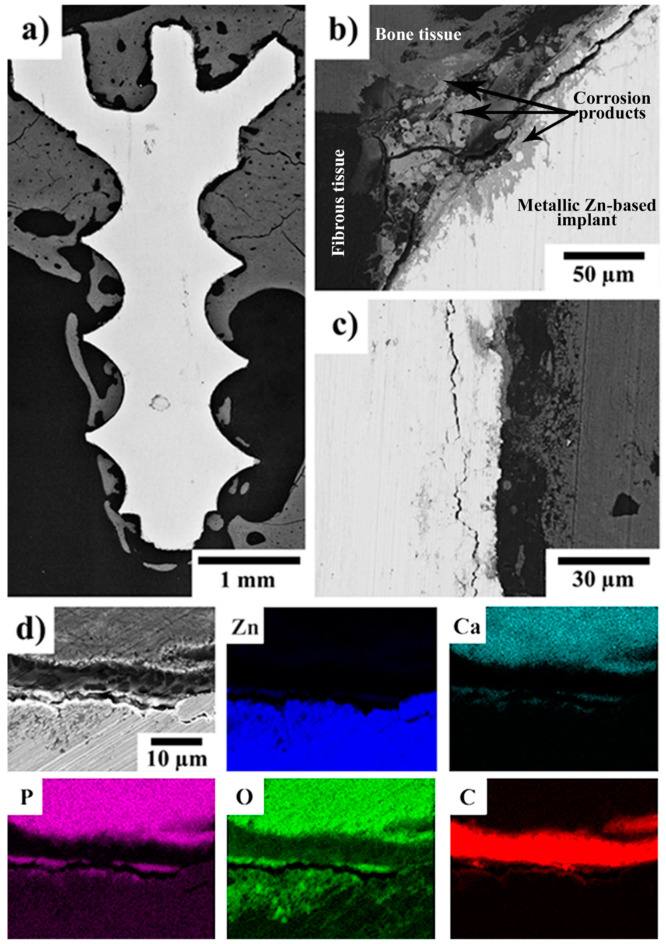
(**a**–**d**) SEM micrographs (Z-contrast) of a Zn-0.8Mg-0.2Sr experimental screw 240 days after implantation in rabbit tibias. The X-ray elemental maps showing the distributions of Zn, Ca, P, O, and C were acquired from the area shown in (**d**); the scale bar is the same as in (**d**). In (**b**) and (**c**), the gray shaded areas can be interpreted as follows: the lightest gray—metallic implant, light gray—corrosion products, dark gray—bone tissue and black/the darkest gray—fibrous tissue.

**Figure 5:**
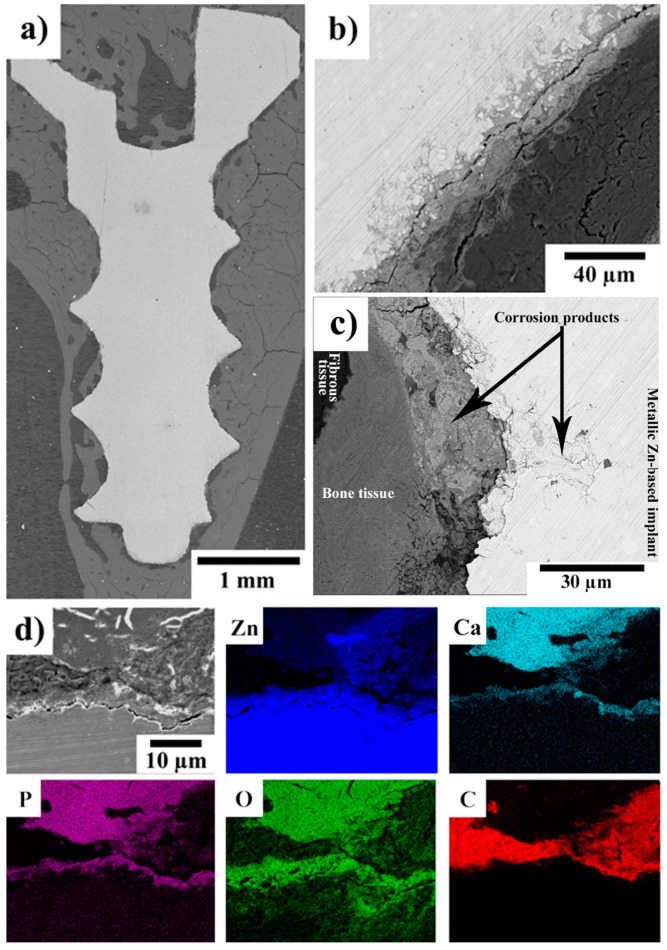
(**a**–**d**) SEM micrographs (Z-contrast) of a Zn-0.8Mg-0.2Sr experimental screw 360 days after implantation in rabbit tibias. The X-ray elemental maps showing the distributions of Zn, Ca, P, O, and C were acquired from the area shown in (**d**); the scale bar is the same as in (**d**). In (**b**,**c**), the gray shaded areas can be interpreted as follows: the lightest gray—metallic implant, light gray—corrosion products, dark gray—bone tissue and black/the darkest gray—fibrous tissue.

Compared to the micro-CT examination, the SEM–EDS observations provided us with more detailed information about the changes of the implant surface and revealed the evolution of the degradation process over time. After 120 days, only a thin (4–5 µm) uniform layer of corrosion products containing Zn, O, P, and Ca was observed on the implant surface (see Figure 4 in our previous invivo pilot study [18]). Locally, oxide-/hydroxide-based corrosion products occurred, penetrating to a depth of 10–12 µm from the surface. After 240 and 360 days, a very similar phosphate-based layer was observed on the surface of the experimental screws, as is visible in Figure 4 and Figure 5. In those cases, the phosphate layer was observed to be 3–4 µm thick. After 240 days, enhanced concentrations of oxygen were locally observed underneath the phosphate-based layer. After 360 days, the oxygen-based layer was significantly more uniform and thicker. Degradation at a depth of approximately 25 µm and 35 µm was observed after 240 and 360 days, respectively. In the case of the screws implanted for 240 and 360 days, we observed cracks parallel with the longitudinal axis of the screw. After 240 days, the cracks were located 15–20 µm from the screw surface. After 360 days, the distance of the cracks from the surface had increased to approximately 20 and 30 µm. Independently of the implantation period, the implants were often surrounded by a layer rich in carbon. As will be shown later, this layer was formed by fibrous tissue. In some locations, direct contact between the solid corrosion products and bone tissue was observed as well (see Figure 4 and Figure 5). In the case of the screws implanted for 240 and 360 days, we even observed Zn-rich areas (corrosion products) fully surrounded by bone tissue. This suggests that some absorption of the screws took place. Unfortunately, the degradation was most likely too small to be distinguished by micro-CT. The fact that the experimental Zn-based alloy was partially dissolved and partially formed by solid corrosion products precludes us from determining exactly the degradation rate and its dependence on time. Despite this, it seems that the degradation rate was smaller after 120 days than after 240 and 360 days. The overall degradation rate was relatively small. Based on the SEM–EDS examinations, it could be estimated that after 360 days it would hardly exceed 100 µm.

**Figure 6:**
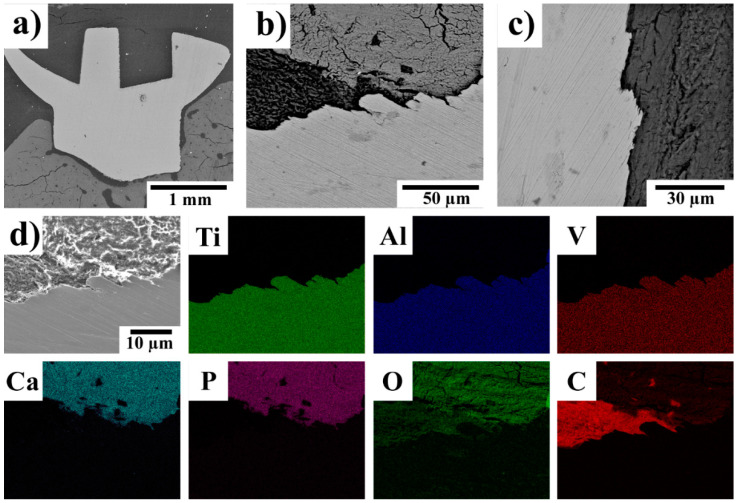
(**a**–**d**) SEM micrographs (Z-contrast) of a Ti-based control screw 360 days after implantation in rabbit tibias. The X-ray elemental maps showing the distributions of Ti, Al, V, Ca, P, O, and C were acquired from the area shown in (**d**); the scale bar is the same as in (**d**). In (**b**,**c**), the gray shaded areas can be interpreted as follows: the lightest gray—metallic implant, dark gray—bone tissue and black/the darkest gray—fibrous tissue.

In contrast to the experimental Zn-based screw, the control Ti-based screw (Figure 6) did not degrade, and most of its surface was surrounded by bone tissue.

### 2.4. Histopathological Examination of Bony Specimens

**Figure 7:**
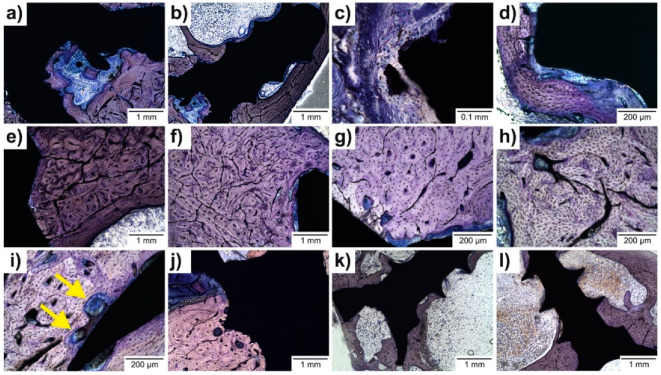
Histological examination of bone specimens (**a**–**f**) 120 days after experimental screw implantation; (**g**,**h**) 240 days after experimental Zn-based screw implantation; (**i**,**j**) 360 days after experimental screw implantation. Mild changes in the experimental screw sharpness, suggesting possible corrosion (**k**,**l**) 360 days after Ti-based screw implantation. Osteon-like cavities—arrows. Figure 7a,c,d are assumed from our previous pilot study [18].

Two to five histological slides from each implant were examined. Selected micrographs are show in Figure 7. The presence of gas bubbles was not detected in any of the examined samples (see Table 3). The structure of the bone in the immediate vicinity of the implant remained, in the vast majority of samples, regular (Figure 7e,g). Rarely, there was some slight disruption of the osteon arrangement—both in the earlier (120 days) and later (240 days) samples (Figure 7f). Osteon-like cavities did not occur in the above-threshold (>3) quantity at the early stage. At the later stage, they were present in a mild form (4–6) in at least one of the preparations in each rabbit (Figure 7i).

Periosteal apposition was significantly expressed in all groups containing an experimental screw (Figure 7i,j) and the control (Figure 7k), and remodeling with bone formation—sometimes lamellar, more often bundle bone (Figure 7d)—was also expressed in most cases. Endosteal remodeling was also present in most cases (Figure 7b), mostly with a slightly lower intensity than periosteal. In individual experimental animals, the intensity of periosteal and endosteal remodeling was mostly similar. In a minority of cases, slight disruption of the osteon arrangement occurred in the immediate vicinity of the implant. This was mild and did not show any dependence on time—it was noticed, rarely, in samples from all time groups. It was not present in the control group (Ti-based screw); the relationship of this finding to the implanted material cannot be ruled out; however, it was so mild (and to some extent, the assessment at this minimal intensity was also subjective) that it did not exclude individual variability of bone formation. Periosteal remodeling developed within 120 days in the experimental alloy group (Group 1) after implantation. In the 240-day group (Group 2), it remained at about the same level. In the 360-day group (Group 3), we noticed a certain decline. It is possible to consider a development, similar to that in the healing of bone fractures, where over time there is also a reduction of structures created in abundance during the early phase of the reparative process. For endosteal remodeling, the maximum change was recorded in experimental Group 1 after 120 days, with a slight subsequent decrease. The evaluated periosteal apposition (as per Raifenrath´s scheme) [30] was expressed and persisted only binarily (present or absent). Peri-implant fibrosis was shown in semiquantitative evaluations to increase over time; a scoring system based on the average value of the semi-quantitatively assessed trait in the group was used for comparison between the groups.

**Figure 8:**
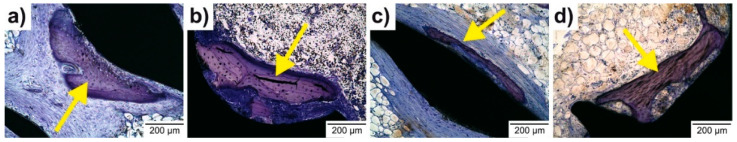
Peri-implant bone formation (arrows), variable thickness of newly formed bone on the surface of the implanted screws (marked by the yellow arrows) (**a**) 120 days after experimental screw implantation, (**b**) 240 days after experimental screw implantation, (**c**) 360 days after experimental screw implantation. (**d**) Control 360 days after Ti-based screw implantation.

Peri-implant bone (PIB) formation was always expressed, but with markedly variable intensity (Figure 8a–d). Bone thickness was neither related to the time duration of the implant nor the metal alloy used. For the quantitative scoring see Table 3.

**Figure 9:**
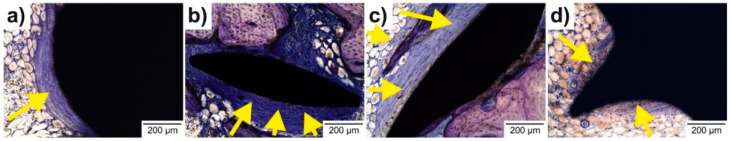
Peri-implant fibrosis (fibrosis is marked by yellow arrows) (**a**) 120 days after experimental screw implantation, (**b**) 240 days after experimental screw implantation, (**c**) 360 days after experimental screw implantation, (**d**) 360 days after Ti-based screw implantation.

Peri-implant fibrosis was expressed in more than 51% of the implant surfaces of the experimental animals, but with variable thickness. The fibrous layer rapidly expanded on the surfaces of the implanted screws in the first 120 days. In the 240- and 360-day experimental groups (Groups 2 and 3) similar thicknesses were observed (Figure 9a–c). The Ti-based screw showed mild to low peri-implant fibrosis (Figure 9d). In individual cases, the fibrous layer had a similar width. Rarely, a focus of thickening was visible in thin peri-implant fibrosis (Figure 9b). Peri-implant fibrosis was objectified by determining the maximum width, morphometrically, as a value (mm) in the evaluation table (Table 3).

**Figure 10:**
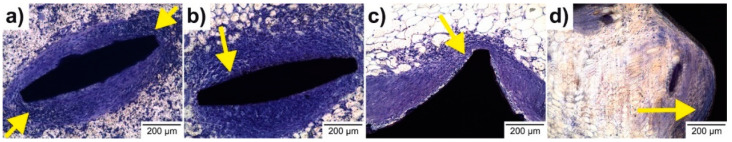
Peri-implant inflammation (marked by yellow arrows) (**a**) 120 days after experimental screw implantation, (**b**) 240 days after experimental screw implantation, (**c**) 360 days after experimental screw implantation, (**d**) 360 days after Ti-based screw implantation.

The inflammatory response in the connective tissue surrounding the implant was moderate to high in intensity (Figure 10a–c) but absent in the control group (Figure 10d). In contrast, the presence of macrophages in the staining used was unremarkable—it was mostly subthreshold (<3 in section) or sparse (3–20 macrophages in section) (Figure 7c). Giant multinucleated cells were found only in one solitary case. The increase in fibrosis over time, compared to the intensity of the inflammatory infiltrate which decreased between Group 2 (240 days) and Group 3 (360 days), can be interpreted as healing with final resting fibrosis, usually accompanied by peri-implant bone formation (PBF). The productive inflammatory phase, combined with the decreasing infiltrate, indicates the compatibility of the material, and is correlated with minimal signs of corrosion of the material and the absence of metallosis.

Beyond the described peri-implant fibrosis, in relation to the corrosion of the material used, no signs of active osteolysis were expressed. In all of the samples of the implanted material, only a very minimal disturbance of the contour sharpness was seen at the level of light microscopy (Figure 7j), which can be considered a sign of resorption. No metallosis, with its corresponding granulomatous reaction, was observed in the environment. There were also no signs of active osteolysis beyond the described peri-implant fibrosis.

### 2.5. Histology Results from Liver, Kidney, and Brain Samples

**Figure 11:**
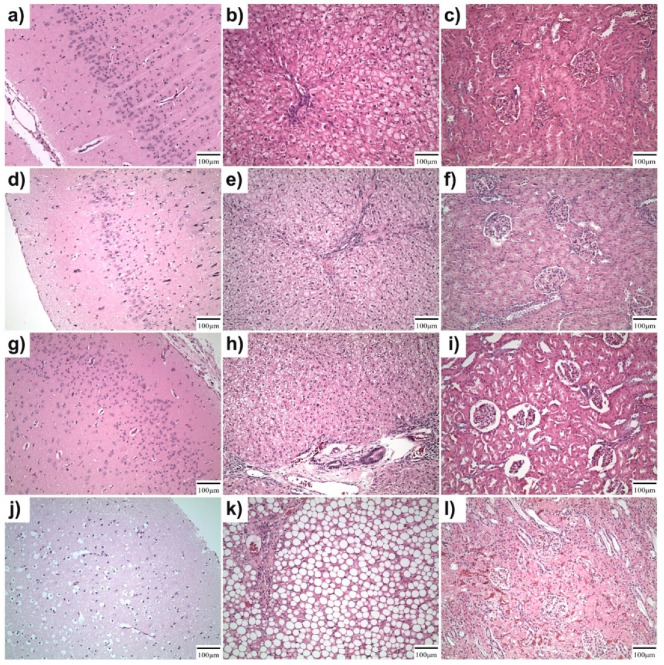
Histological analysis of vital organs, brain (left column), liver (middle column), and kidney (right column) (**a**–**c**) 120 days after experimental screw implantation, (**d**–**f**), 240 days after experimental screw implantation, (**g**–**i**) 360 days after experimental screw implantation, (**j**–**l**) 360 days after Ti-based screw implantation. No detectable changes between experimental screw groups and Ti-based screws used as a control. (**k**) Massive hepatic steatosis visible in the liver occurred 360 days after Ti-based screw implantation.

The kidneys, livers, and brains from the experimental rabbits were examined histologically at 120, 240, and 360 days after screw implantation. The same specimens were also analyzed in the control group, which had Ti-based screws implanted. In the three experimental groups (groups 1–3) (*n* = 9), implanted with the experimental alloy, no histological changes were found, confirming no toxic damage to their kidneys, liver, or brain due to the absorbable Zn-based alloy after 120, 240, and 360 days (Figure 11a–l). The architecture and cytomorphological characteristics of the parenchymatous organs did not show any regressive changes that might be related to absorbable metal alloys being used. In the control group (*n* = 3) the abovementioned vital organs also exhibited normal structures. Massive hepatic steatosis occurred a total of three times: once in the rabbit with the experimental alloy screw euthanized 360 days after implantation, and twice in the control rabbits with Ti-based screws (Figure 11k). When taking in to account its described morphological characteristics and occurrence, it appears highly unlikely to be related to the implant material. No other organs showed any signs of steatohepatitis and there were no toxic changes in the parenchymatous organs.

### 2.6. Results of Organ Toxicity Analysis

Utilizing the Page trend test, we analyzed whether the content of the Zn, Mg, or Sr elements varied over time in each of the experimental groups. The content of Mg_kidney_ and Mg_liver_ decreased statistically over time (*p*-value = 0.03241). The mean content of magnesium in the kidneys decreased from 0.177 mg/g after 120 days to 0.159 mg/g after 360 days of exposure to the implant. The livers of the rabbits, 120 days after implantation, contained 0.179 mg/g of magnesium. This content decreased to a mean value of 0.160 mg/g after 360 days. The content of magnesium in the brain did not differ statistically over time (*p*-value = 0.2778) with mean values of 0.144 and 0.141 mg/g after 120 and 360 days, respectively. The content of zinc in the brain and kidneys of the experimental groups was also not statistically different (Zn_brain_; 120 days = 10.4 µg/g, Zn_brain_, 360 days = 8.9 µg/g, *p*-value of 0.5556, and Zn_kidney_;120 days = 24.3 µg/g, Zn_kidney_, 360 days = 21.3 µg/g, *p*-value of 0,1528). In contrast, the zinc content in the liver significantly decreased over time between the 120-day and 360-day groups (from 31.6 µg/g to 22.8 µg/g; *p*-value = 0.03241). The strontium levels in the brain, kidneys, and liver did not statistically differ over time (Sr_brain_: *p*-value = 0.4444, Sr_kidney_: *p*-value = 0.5556, Sr_liver_: *p*-value = 0.5556). The mean values for the amount of strontium in the organs were very low; for the brain, they were on the order of tenths of µg/g and in the kidneys and liver, they were on the order of hundredths of µg/g. Using the Mann–Whitney test, we statistically analyzed the content of each individual element (Zn, Mg, Sr) in the brain, kidneys, and liver of the experimental group implanted for 360 days (Group 3) and the control group (Group 4), assessing the differences between the medians. We did not find any statistically significant differences between the organs of the rabbits implanted with the experimental Zn-based alloy and the control Ti-based alloy. Based on our results, we can summarize that the content of Zn, Mg, and Sr in the organs in the experimental groups (Group 1–3) did not increase over time. Furthermore, in some of the groups the content of the elements actually decreased over time: Mg_kidney_, Mg_liver_, and Zn_liver_. When comparing the median concentrations of Zn, Mg, and Sr in the brain, kidney, and liver of the experimental and control groups, no statistically significant difference was found.

## 3. Discussion

Age related fractures are projected to increase in the US to over 3 million fractures per year by 2025 [1]. Steel or titanium alloy plates, screws, nails, and meshes are the gold standard for fracture treatment [31]. According to published statistics, the global market for fracture fixation devices is estimated to be worth USD 5.5 billion [32]. The use of titanium or steel materials in the treatment of fractures brings with them many disadvantages: (i) thermic sensitivity [33], (ii) the tactile sensation of the plates and screws [34,35], (iii) the restriction of bone growth [34,36], and (iv) stiffness causing stress shielding of the underlying bone [34,37]. In 2018, 176,257 operations for implant removal were performed in Germany [38]. This means that the metallic material was removed in about 80% of fractures treated by osteosynthesis [38,39]. The US reports similar numbers [40]. In Germany, in 2007, it was estimated that the expenses from these procedures exceeded EUR 430 million per year [34,41]. Minimizing the number of operations would correspondingly decrease patient morbidity and bring about further significant financial savings. The use of absorbable materials based on polyhydroxyl acids, polymers and copolymers, for example, PLLA, poly-d-lactic acid (PDLA), or polyglycolic acid (PGA), have been used to try and solve the disadvantages of permanent metallic implants [42]. Their low strength disposes them to use only in some skull fractures, where the mechanical loads are small [42]. The development of an absorbable metallic material would combine the benefits of inert metals and absorbable polymers. It can result in appropriate mechanical properties [43] and absorption of the implant after it has fulfilled its’ mission of fracture healing.

As shown and discussed in our previous study [43], the experimental alloy fulfils all the basic mechanical criteria required for bone fixation (tensile yield strength > 230 MPa, ultimate tensile strength > 300 MPa and elongation to fracture > 15%–18%) [44]. All of the elements (Zn, Mg, and Sr) play an important role in bone metabolism and in appropriate amounts, can significantly enhance the healing process [45,46,47,48]. Investigation of the in-vitro cytotoxicity of the experimental alloy showed appropriate cytocompatibility and even enhanced antibacterial activity [43]. Based on those data, the investigated alloy can be considered as a promising candidate for the fabrication of bone implants.

The analyzes performed in this study have shown that the content levels of Zn, Mg, and Sr in the brain, liver, and kidneys of the experimental groups (Group 1–3) did not change significantly over time. Moreover, we detected that the content levels of Mg in the kidneys, and of magnesium and zinc in the livers of the experimental groups, decreased over time. There was no statistical difference in the Zn, Mg, and Sr content of the organs between the experimental and control groups. The results indicated that the decrease over time of the content of selected elements (Mg and Zn) in individual organs (kidneys and liver) was most likely connected with the aging of the rabbits.

The possibility of implant toxicity was studied histologically in the vital organs. We analyzed specimen of the brain, liver, and kidneys from all the animals in the experimental and control groups. In the experimental groups, the brain and kidneys exhibited a normal histological appearance. Massive hepatic steatosis occurred a total of three times: once in the rabbit with the experimental alloy screw euthanized 360 days after implantation, and twice in the control rabbits with Ti-based screws (Figure 11k). There are two different types of liver steatosis (i) alcoholic and (ii) non-alcoholic fatty liver disease (NAFLD) [49]. In recent years, an increase in calorific intake along with a reduction of physical activity have led to an increase in obesity and a parallel increase in the prevalence of non-alcoholic fatty liver disease in humans [49]. Steatosis was only observed in the animal groups with the longest experiment time. Hepatic steatosis in alcoholics is caused by the toxic effects of alcohol on liver cells. Similarly, we could speculate that the liver steatosis in our experiment was caused by the toxic effect of the alloy elements on the liver. Although we cannot exclude this, the involvement of one animal from the experimental group and two from the control group suggests that there is no relationship between the experimental alloy and steatosis. The most likely reason for liver steatosis was actually a combination of the long period of experimentation, a high calorific intake, and the low physical activity of the rabbits.

The implant–tissue interaction was studied histologically and was evaluated according to the method of Reifenrath et al. [30]. No gas bubbles were observed histologically. This correlates with the results of Hybášek et. al. [27]. They proved with a set of electrochemical measurements that the production of hydrogen as an accompanying event of the corrosion process of the investigated Zn-based alloy was unlikely [27]. This absence of hydrogen gas formation is a big advantage over Mg-based absorbable materials. Hydrogen production is often accompanied by inflammatory reactions and by the formation of cavities encapsulated by fibrous tissue [50]. Cortical bone is formed in concentric ring-like structures, called osteons [51]. Osteons contain a central canal, called the Haversian canal, which surrounds the blood vessels within the bone [51]. Osteocytes reside in lacunae within the bone matrix forming a cellular network known as the osteocyte lacunar-canalicular system [51,52]. The osteocyte lacunar-canalicular network needs to be re-established after successful bone healing [51]. Osteon-like cavities did not occur in the above-threshold (>3) quantity at the early stage. At the later stages (240 and 360 days), they were present in a mild form (4–6) in at least one of the preparations from each rabbit (Figure 7i—arrows). The number of osteon-like cavities increased from 120 days to 240 days in the experimental group and were also comparable to the number of osteon-like cavities in the control group (Table 3). We can conclude that the increasing number of osteon-like cavities from the 120 days group to the 240 days group is related to the bone maturation process. The morphology of the bone adjacent to the experimental screw had a normal biological appearance and was comparable with the control group. The outer membrane covering the bone is known as the periosteum [53,54]. The membrane lining the wall of the bone marrow cavity is known as the endosteum [54]. Periosteum and endosteum contain cells (osteoblasts, osteoclasts, and osteoprogenitor cells) required for bone development and remodeling [53]. The periosteum and endosteum are essential for growing, fracture healing, and remodeling [53,55]. Any irritation or disruption to the underlying bone will cause a periosteal reaction and result in new periosteal bone deposition [56] and remodeling. In our study, periosteal bone apposition was expressed significantly in all the groups with the experimental screws (Figure 7i,j) and in the control group (Figure 7k). Endosteal remodeling was also present in most cases (Figure 7b), mostly with a slightly lower intensity than periosteal remodeling. In each individual experimental animal, the intensity of periosteal and endosteal remodeling was similar. In our opinion this remodeling process mimicked physiological remodeling as the experimental and control groups had similar periosteal and endosteal remodeling results. Periosteal bone formation occurs throughout life [57]. Our data shows that the periosteal apposition had a similar intensity in all experimental and control groups (Table 3). This could support our hypothesis that this natural process was not disturbed by the experimental alloy screw. Osseointegration was originally defined as a direct structural and functional connection between living bone and the surface of a load-bearing implant [58]. Peri-implant bone (PIB) formation was always expressed histologically, but with markedly variable intensity (Figure 9a–d). Bone thickness was completely independent of both the time of the implant and the metal alloy used (Table 3).

Bone–implant contact (BIC) refers to the amount of the implant surface boundary in direct contact with new bone [59]. The BIC was measured by means of micro-CT. All the evaluated implants were in direct contact with their surrounding bone and did not show any signs of any adverse reaction. The thickness of the bone surrounding the implants was higher in comparison to the average cortical thickness, which proves biocompatibility of the tested materials. The mean BIC values obtained by the micro-CT analyses are listed in Table 1. The were no significant differences in BIC between the experimental and control groups (Mann–Whitney paired test, *p* = 0.05). In practice, osseointegration means that there is an anchorage mechanism whereby nonvital components can be reliably and predictably incorporated into living bone and that this anchorage can persist under all normal conditions of loading [60]. Peri-implant bone formation and BIC are very good signs of osseointegration. We can conclude that peri-implant bone formation histologically and BIC (evaluated by micro-CT) demonstrate osseointegration and thus good biocompatibility to the adjacent bone. If an intrabony implant is not osseointegrated but instead surrounded by fibrous tissue, it is called fibrointegration [61,62]. Peri-implant fibrosis was observed in more than 51% of the experimental alloy surface, but with variable thickness. The fibrous layer expanded on the surface of the implanted screws very rapidly during the first 120 days. In the 240- and 360-day experimental groups (Groups 2 and 3) we observed the thickness to be similar (Figure 9a–c). Peri-implant fibrosis was more prominent around the experimental implants. The Ti-based screw showed mild to low peri-implant fibrosis (Figure 9d). The thickness of the fibrous layer was measured by histological morphometry in millimeters (Table 3). There was not a significant difference in the thickness variation around experimental screws. Other studies have confirmed a high presence of fibrous tissue found around rapidly degrading Zn-based and Mg-based implants [63,64]. Severe localized corrosion led to the production of a thick fibrotic layer surrounding the implants [29]. In terms of corrosion behavior, it is generally acknowledged that iron-based metals corrode slowly, magnesium-based metals corrode rapidly, and zinc-based metals corrode moderately [65]. After decades of acknowledging that metal implants must be corrosion resistant, nowadays, corrosion is seen as an advantage [65]. Absorbable metals are expected to corrode gradually in vivo by generating an appropriate host response and then dissolve completely upon assisting tissue healing [66]. In order to achieve optimal osseointegration, resistance to corrosion is important [67,68,69,70]. Since 1977, titanium has been recognized as the gold-standard for high-quality of osseointegration [58]. Yang et al. reported in 2020 that the BIC depends on the degradation/corrosion behavior of implants [23]. Uniform corrosion with appropriate degradation rates resulted in improved BIC, whereas severe localized corrosion led to the production of a thick fibrotic layer surrounding the implants [29].

Absorbability requires a reaction from the tissues adjacent to the bone. This response should not be aggressive thus not producing osteolytic changes to the bone or a significant inflammatory reaction. However, corrosion is not the only explanation for peri-implant fibrosis/fibrointegration. The implantation of any foreign material into the body will lead to an inflammatory and fibrotic process—the foreign body reaction (FBR) [62]. The foreign body reaction can be defined as the tendency of a living organism to isolate itself against a foreign body with a layer of connective (fibrous) tissue. This first stage of this reaction is based on inflammation. The inflammatory reaction in the surrounding tissues of the experimental groups was of a moderate to high intensity (Figure 10a–c) but was absent in the control group (Group 4) (Figure 10d). We observed a high density of inflammatory cells in Group 2 after 240 days. The reasoning for this remains unclear. In contrast, the presence of macrophages was rarely seen in any of the groups (Figure 7c). The hallmark of a foreign body reaction is the fusion of macrophages into giant multinucleated cells, which could be seen on the surface of the implants [62]. Giant multinucleated cells were observed in only one solitary case (Table 3). Metallosis refers to deposition of metallic debris into the periprosthetic soft tissues [71]. No metallosis with its’ granulomatous reaction was observed. Osteolysis (i.e., active resorption of the bone matrix) and the formation of a thick fibrous layer between the implant and bone are indicative of poor biocompatibility [72]. There were also no histological signs of active osteolysis beyond the described peri-implant fibrosis. Based on the aforementioned data the peri-implant fibrosis is not based on a foreign body reaction. We did not detect any signs of osteolytic changes around the experimental screws. The implanted material showed no disturbance in the sharpness of the contours in any of the samples at the level of light microscopy (Figure 7j) which is considered a sign of resorption.

The results of the X-ray and micro-CT examinations did not reveal any adverse effects from the experimental Zn-based implant on the quality of the surrounding bone. Based on the aforementioned data, the alloy can be considered as potentially safe candidate for implantology.

Investigation of the implant–bone interface by SEM–EDS allowed us to examine the interface on a microscopic scale and from the viewpoint of the chemical composition. Independently of the implantation period, a thin and compact phosphate-based layer (3–5 µm) was found on the surface of the implanted Zn-based screws. Calcium and zinc were detected in this layer, which suggests the formation of a complex or a mix of Ca/Zn phosphates in in-vivo conditions. The formation of such a layer can be attributed to the interaction of Zn^2+^ ions, released by the dissolution of the experimental alloy [73], with the body fluids containing Ca^2+^, H_x_PO_4_^(3−x)−^, and other ions. These formed phosphates subsequently precipitate on the implant surface [12]. The formation and precipitation of these phosphates is also influenced by the presence of proteins, which can form complex compounds with zinc and ions from the solution. Those complex compounds subsequently enhance the phosphate precipitation [28,74]. Based on the results presented in our previous studies [27,28], we hypothesize that the phosphate based layer is formed in the early stage (up to several days) after implantation. The formation of the phosphate layer is beneficial. As was found by Su et. al. [12], a surface layer of zinc phosphate (ZnP) enhances the cytocompatibility and hemocompatibility of Zn-based materials. They also reported an enhanced antibacterial activity of the ZnP coated samples. The antibacterial activity of zinc phosphate was described also by Chou et. al. [75], who studied the influence of ZnP coatings on the behavior of organic guided bone regeneration (GBR) membranes. Thus, the formation of the ZnP layer with its predictable antibacterial activity, which was observed in our study, could be a reason why only a very limited inflammatory reaction was observed.

Corrosion was observed under the phosphate layer, particularly after 240 and 360 days. With increasing time, the localized process was observed more frequently and at deeper levels within the implants. The localized corrosion can most likely be ascribed to the multiphase composition of the alloy [43]. The intermetallic phases are less noble and corrode preferentially [27]. The SEM–EDS analyses suggest that the local corrosion damage was accompanied by the formation of zinc oxide/hydroxide; however, the formation of alkaline zinc chlorides cannot be excluded as well [27]. The formation of cracks could be attributed to the presence of intermetallic phases. As was shown in our previous studies [28,43], the intermetallic phases were aligned in rows parallel with the extrusion direction and the distance between the rows was in the order of tens of micrometers. A similar distance of the cracks to the surface of the corroded implants was observed in this work (see Section 3.3). When the corrosion medium (body fluids) penetrated to the row of intermetallic phases, the intermetallic phases corrode preferentially forming cracks. As a consequence, metallic particles can be released from the implant [76]. This was also observed by SEM and SEM–EDS analyses after 240 and 360 days of implantation (Figure 4b and Figure 5d) in which Zn-rich particles were surrounded by tissue. No such event was observed after 120 days of the implantation period [18]. The SEM observations suggest that new tissue spread through the cracks, which were formed by the corrosion process. Consequently, Zn-rich particles were embedded in the tissue and consequently were ingrown within the tissue. It was found that the released particles did not cause any adverse issues and that they were later incorporated into the newly formed bone tissue. The SEM–EDS mapping also suggests that the released particles were based on oxides/hydroxides or phosphates but were not of a metallic character. This means that they consisted of the corrosion products of zinc. Such degradation behavior can be considered beneficial for applications such as absorbable bone implants. As we did not observe metallosis, encapsulation of the particles by fibrous tissue, or any inflammatory reaction around those particles, we can therefore assume that they will eventually become ingrown within the bone tissue and will be slowly absorbed over time. The risk of the stress-shielding effect or other undesired long-term issues caused by those particles should be minimal, especially when compared with metallic screws. For example, a significantly lower ion release can be expected from the inorganic compounds, with their low solubility, than the release from the metallic zinc alloy. This minimizes the risks connected with local toxicity [15,28,43].

Although we did not measure the zinc concentration in serum or blood in the vicinity of the implants, we can suppose, that the risk of the accumulation of Zn^2+^ ions near the implant to high and dangerous concentrations is negligible for two reasons: (i) the exchange of bodily fluids should eliminate the accumulation of the leached ions [3,14]; and (ii) the excess of free Zn^2+^ ions would result in chemical reactions with body fluids and the subsequent formation of solid compounds (phosphates, etc.), which would precipitate on the surface of the implant forming a layer of solid corrosion products [12,28,77]. As we have mentioned above, we did not observe any thrombosis or metallosis, so the release of metallic particles or particles of corrosion products into bloodstream most likely did not take place.

The SEM–EDS observations revealed the degradation and absorption of the investigated material under in-vivo conditions, but the degradation/absorption rates were slow. Ideally, implants should maintain their mechanical integrity for a minimum of 6 months and then be fully absorbed in 1–2 years [78]. This means that the investigated screws with a diameter of 2 mm should ideally lose at least one half of their cross-sectional diameter by 360 days. We were not able to precisely determine the degradation losses, because the pixel resolution/voxel of micro-CT is only about 15 μm. The corrosion products and surface degradation detected by SEM–EDS were smaller than the size of one voxel, so they could not be accurately detected by micro-CT. In order to detect any structural defects in our samples, such as the reported cracks, their size would need to be at least 2–3 voxels to be reliably detected—which equates to 30–40 μm. Volume evaluation was performed. Some change in terms of volume decrease was observed (Table 2) but this was not statistically significant. Nevertheless, the SEM–EDS analyses suggest that the degradation loss was probably less than 100 µm after 360 days, which is less than desired. In-vivo degradation rates ranging between several tens and hundreds of micrometers have also been reported in other studies dealing with Zn-based alloys intended for bone implants [3,73,79,80].

## 4. Materials and Methods

### 4.1. Material Preparation

In this study, we investigated an extruded Zn-0.8Mg-0.2Sr (wt.%) alloy. We chose this alloy, as in our previous studies [27,28,43] we found this alloy to possess promising behavior (mechanical, corrosion and biological) for the fabrication of bone implants. The investigated alloy was prepared by melting appropriate amounts of the pure elements (Zn, Mg and Sr) in a graphite crucible in air atmosphere. The melting was performed at 520 °C. After sufficient homogenization the melt was poured into a steel mold with a diameter of 50 mm and a height of 400 mm. The as-cast ingot was annealed at 350 °C for 24 h to homogenize its chemical and phase composition. After the annealing, the ingot was quenched in water. Subsequently, billets with a diameter of 30 mm and length of 35 mm were machined and extruded at a temperature of 200 °C and an extrusion ratio of 25:1. A detailed description of the preparation procedure of the alloy is listed in our previous study [43].

The exact chemical composition of the alloy was checked via atomic absorption spectrometry using an Agilent 280FS AA spectrometer with flame atomization (Agilent, Santa Clara, CA, USA). For this analysis, samples from several locations of the extruded rod were taken, dissolved in nitric acid, and diluted by deionized water to a concentration suitable for the analysis. The average chemical composition was as follows: 0.83 wt.% of Mg, 0.17 wt.% of Sr and 99 wt.% of Zn.

The experimental screws were prepared from the extruded rod by CNC machining using a CNC Fanuc Robodrill α-T21iFa machine (Fanuc, Tsukuba, Japan). The shape and dimensions of the experimental screws were inspired by “mini-maxillofacial” screws; however, the design was slightly adjusted to withstand the forces necessary for implantation and to better fit into the rabbit tibia. The design of the screw used is shown in more detail in our previous paper [18].

### 4.2. Animals

We used 12 male New Zealand rabbits (Velaz, Prague, Czech Republic) with body weights between 750–850 g. We only used males in our study to minimize the effect of hormone levels on the variability of healing and bone regeneration [81]. This study was performed in accordance with the European Communities Council Directive of 24 November 1986 (86/609/EEC) regarding the use of animals in research and was approved by the Ethical Committee of the Ministry of Education Youth and Sports of the Czech Republic on the 9 May 2018 (protocol code: MSMT—7025/2018-6). For ethical reasons, we only used 12 animals in order to minimize the number of animals used in the study.

### 4.3. Experimental Groups

Twelve experimental rabbits were randomly divided into four of the following groups: 3 groups with the experimental alloy (Zn-0.8Mg-0.2Sr) screw implanted in their tibial bones (Groups 1–3) (n = 3). The last group (Group 4) (n = 3) were treated with standard Ti-based screws which have EU approval for human use. This screw was a “mini-screw” with a diameter of 2mm, made from a Ti-6Al-4V ELI alloy (Jeil Medical, Seoul, Republic of Korea) in conformity with ASTM F136. The rabbits were euthanized after 120 days (Group 1), 240 days (Group 2), and 360 days (Group 3 together with Group 4—the control group). Details of how the euthanasia was performed is listed in our previous work [18]. Bone specimens containing the experimental screws, along with specific internal organs (kidneys, liver, and brain) were harvested from the euthanized animals and fixed in a 4% formalin solution to be processed for examination in the same way as in our previous study [18].

### 4.4. Surgical Procedure

The screws were implanted into rabbit tibiae under general anesthesia at a certified operating veterinary theatre in the exactly same way as we have described in our previous study [18].

### 4.5. Methods of Radiographic Examination

X-ray examination of all experimental rabbits was performed under general anesthesia 120, 240, and 360 days after implantation. After the induction of anesthesia utilizing 5% isoflurane in air (flow 300 mL/min), two projections of the rabbit tibia were taken, one projection perpendicular to other. The X-ray examination was performed using an In-Vivo Xtreme BI 4MP equipment (Bruker BioSpin, Rheinsetten, Germany).

### 4.6. Methods of Micro CT

Bone specimens (n = 12) containing inserted implants were scanned using a desktop micro-CT SkyScan 1272 (Bruker micro-CT, Kontich, Belgium). Micro CT analysis was performed using the same protocol as was described in our pilot study [18]. Prior to 3D analysis of the implant, parametric data (volume and surface values) were image-processed in order to improve the signal-to-noise ratio and subsequently binarized. Data processing was optimized using TeIGen software (Teigen Technologies, Oslo, Norway) [82]. Bone-implant contact (BIC) was quantified through manual measurements of 2D cross-sectional images of each specimen (Equation (1)):

(1) $$\mathrm{BIC}=\frac{\mathrm{implant}\;\mathrm{perimeter}\;\mathrm{in}\;\mathrm{contact}\;\mathrm{with}\;\mathrm{bone}}{\mathrm{implant}\;\mathrm{perimeter}\;}$$

Bone contact was only evaluated in the cervical region as the apex position of the implants varied among the specimens. The heads of the screws were excluded from this evaluation.

### 4.7. Bone–Implant Interface Preparation

For the examination and histological analysis of the solid corrosion products, thin (≈60 µm) longitudinal sections were prepared by a standardized procedure, the details of which are described in our previous study [18].

### 4.8. Examination of the “Implant–Bone” Interface

The implant–bone interface was observed using a scanning electron microscope FEI Quanta 3D FEG (ThermoFisher Scientific, Waltham, MA, USA) equipped with an EDAX Apollo 40 energy dispersive spectrometer (Ametek, Berwyn, PA, USA) (SEM–EDS). The obtained data were used to evaluate the excess and uniformity of the corrosion attack. The chemical composition of the solid corrosion data were also estimated using the acquired data.

### 4.9. Histological Analysis Method Used for Bone Specimen

The bone reaction—its morphology, and the presence of fibrous tissue and cells—was studied and evaluated in histopathology specimens using a semiquantitative scoring system of parameters according to Reifenrath et al. 2011 [30]. The healing process of the tested materials in the artificially created holes and grooves was examined by toluidine blue stained slides with optical microscopy using a Nikon Eclipse 80i microscope (Nikon Instruments Inc., Melville, NY, USA), Jenoptik camera (Jenoptik, Jena, Germany) and an image analysis system from NIS Elements, Nikon AR (Nikon Instruments Inc, Melville, NY, USA). The evaluated features were: gas bubble formation, overall bone structure, bone cavities, periosteum remodeling, endosteal remodeling, periosteum apposition, peri-implant bone formation, peri-implant fibrosis, lymphoplasmocellular reaction, the presence of macrophages, and giant cells [30]. Two to five histological slides from each implant were examined. We monitored for signs of possible damage and bone tissue response at the level of: (i) the periosteum, (ii) endosteum, (iii) implant bone contact (BIC) [83,84], (iv) connective tissue formation, (v) inflammatory response, and (vi) ossification in the newly formed connective tissue [30] as we described in our previous study [18].

### 4.10. Histopathological Method Used for Processing the Parenchymal Organs

The organs of the euthanized rabbits were fixed in buffered formalin. First, the organs were examined macroscopically, without any pathological changes observed. Subsequently, standard hematoxylin and eosin staining was used. Kidney, liver, and brain specimens from all animals were examined, looking for any possible toxicity resulting from the implanted material.

### 4.11. Analysis of Systemic Toxicity in Vital Organs

Liver, kidney and brain specimens were analyzed for their content of Strontium, Magnesium and Zinc as per the description in our previous study [18]. The content of Sr and Zn elements were analyzed by Inductively Coupled Plasma Mass Spectrometry (ICP-MS) on an ELAN DRC-e instrument (Perkin Elmer, Waltham, MA, USA). Magnesium content was analyzed by F-AAS Flame Atomic Absorption Spectrometry on an AAnalyst 400 (Perkin Elmer, Waltham, MA, USA).

The concentrations of the alloy elements (Zn, Mg and Sr) were analyzed in the brain, liver, and kidney after 120, 240, and 360 days in the experimental groups (Groups 1–3 respectively) and at 360 days in the control group (Group 4). The obtained results were statistically investigated. We used the Page trend test and the Mann–Whitney test for statistical analysis. We considered a *p*-value < 0.05 to be statistically significant. Statistical analysis was performed on R 3.6.3 software (R-foundation, Vienna, Austria) using the “DescTools” and the “Coin” package.

## 5. Conclusions

In this study, a Zn-0.8Mg-0.2Sr alloy and a Ti-based control were successfully implanted into 12 rabbits and monitored for 360 days. This represents a longer experiment compared with other published studies. We did not detect any local or organ toxicity from the experimental alloy. The alloy showed good biocompatibility with a low inflammatory response. It was proven histologically that the experimental alloy presents a promising prospect for future clinical studies of Zn-based materials. The degradation of the alloy was connected with its transformation into solid corrosion products and the release of particles, which were fully incorporated into new tissue. For applications in bone surgery, the degradation rate should ideally be enhanced. Despite this drawback, the presented study clearly shows the great potential of the use of Zn-based alloys in bone surgery.

## Figures and Tables

**Table 1:** Bone–implant contact (BIC) values after various times of implantation.

Test				Control (Ti-Based Alloy)
**Experimental Time**	120 days	240 days	360 days	
**BIC**	0.22 ± 0.02	0.32 ± 0.11	0.33	0.27 ± 0.06

**Table 2:** Values of implant volume and surface in different time periods.

	Implantation Time (Days)		
	120	240	360
**Implant Volume (mm^3^)**	12.40 ± 0.50	12.25 ± 0.23	12.64 ± 0.43
**Implant Surface (mm^2^)**	57.16 ± 0.57	56.55 ± 2.80	58.45 ± 1.44

**Table 3:** Evaluation of the healing process according to the parameters of the histology specimens; bone morphology and cells were assessed with a semi-quantitative scoring system, using the method described by Reifenrath et al. [30]. Note: NA = not applicable, NI = no implant visible, NC = not applicable as screw in compacta.

Parameter	Score	Interpretation	Group 1 [18]				Group 2				Group 3				Control Group 4		
Gas bubbles	0	No	0	0	0	0	0	0	0	0	0	0	0	0	0	0	0
	1	Yes															
Overall impression of bone structure (BS)	0	smooth	0	0					0	0	0	0	0	0	0	0	0
	1	Irregular			1	1	1	1									
Bone cavities (BC)	0	≤3 osteonlike cavities	0	0	0	0	0			0	0		0			0	
	1	4–6 osteonlike cavities or ≤10 smaller						1	1			1		1	1		1
	2	7–10 osteonlike cavities or 11–20 smaller															
Periosteal remodeling (PR)	0	No			NA	1					0		0	0			
	1	≥1/4 periosteal bone 1 osteon thick	2	2			1	1	1	1		1					
	2	≥1/4 periosteal bone 2 osteon thick													2	2	2
	3	≥1/4 periosteal bone 3 osteon thick															
Endosteal remodeling (ER)	0	No		0		NC					0						
	1	≥1/4 endosteal bone 1 osteon thick					1	1	1	1			1				
	2	≥1/4 endosteal bone 2 osteon thick			2							2		2	2	2	2
	3	≥1/4 endosteal bone 3 osteon thick	3														
Periosteal aposition (PA)	0	No			NA												
	1	Yes	1	1		1	1	1	1	1	1	1	1	1	1	1	1
Peri-implant bone formation (PIF)	0	No															0
	1	Yes	1	1	NI	1	1	1	1	1	1	1	1	1	1	1	
Peri-implant fibrosis (PF)	0	No															
	1	≤25% implant surface													1		
	2	25–50% implant surface														2	2
	3	≥51% implant surface	3	3	NI	3	3	3	3	3	3	3	3	3			
	[mm]	max. thickness	0.1	0.1	0.05	0.04	0.23	0.15	0.14	0.14	0.12	0.15	0.22	0.18	0.05	0.05	0.05
Lymphoplasmacellular reaction (LYM)	0	<30 cells per section	0	0	0	0					0	0	0	0		0	0
	1	30–50 cells per section													1		
	2	51–100 cells per section							2								
	3	>100 cells per section					3	3		3							
Macrophages (MPH)	0	<3 cells per section		0	0	0	0	0	0	0		0		0	0	0	0
	1	3–20 cells per section	1								1		1				
	2	>20 cells per section															
Giant cells (GC)	0	No	0	0	0	0	0	0	0	0	0	0	0	0	0	0	0
	1	1–10 cells per section															
	2	>10 cells per section															
Interface—features of material corosion	0	No	0	0	0	0	0	0	0	0	0	0	0	0	0	0	0
	1	Yes															

## Data Availability

The data presented in this study are available on request from the corresponding author.
